# A nanocomposite competent to overcome solubility and permeation issues of capsaicin and thiocolchicoside simultaneously in gout management: Fabrication of nanocubosomes

**DOI:** 10.1016/j.jsps.2024.102050

**Published:** 2024-03-24

**Authors:** Barkat Ali Khan, Falak Naz, Ali Alqahtani, Muhammad Khalid Khan

**Affiliations:** a Drug Delivery and Cosmetic Lab (DDCL), Gomal Centre of Pharmaceutical Sciences, Faculty of Pharmacy, Gomal University, D.I.Khan, 29050, Pakistan; b Department of Pharmacology, College of Pharmacy, King Khalid University, Abha 62529, Saudi Arabia

**Keywords:** Transdermal delivery, Nano-cubosomes, Capsaicin, Thiocolchicoside, Gout

## Abstract

This study aimed to formulate nano-cubosomes (NCs) co-loaded with capsaicin (CAP) and thiocolchicoside (TCS) to enhance their bioavailability and minimize associated potential side effects through transdermal delivery alongside their synergistic activity. Twenty seven (27) nano-cubosomal dispersions were prepared according to Box-Behnken factorial design and the effect of CAP, TCS, glyceryl mono oleate (GMO) and poloxamer 407 (P407) concentrations on particle size, polydispersity index (PDI), zeta potential, and entrapment efficiency were assessed. The results revealed that the optimized formulation exhibited a mean droplet size of 503 ± 10.3 nm, PDI of 0.405 ± 0.02, zeta potential of −10.0 ± 1.70 mV and entrapment efficiency of 86.9 ± 3.56 %. The *in vivo* anti-inflammatory effect of optimized formulation was studied in rats by injecting carrageenan to induce edema. The results of *in vivo* study showed that transdermal application of nano-cubosomes co-loaded with CAP and TCS significantly (p value < 0.05) improved carrageenan induced inflammation compared with standard treatment. The analgesic activity of optimized formulation was evaluated in rats by using Eddy’s hot plate method. The findings of analgesic activity illustrated that the analgesic effects exhibited by test formulation may be associated with increased licking period and inhibition of prostaglandins level. In conclusion, the transdermal application of NCs co-loaded with CAP and TCS may be a promising delivery system for enhancing their bioavailability as well as synergistic analgesic and anti-inflammatory activity in gout management.

## 1 Introduction

Transdermal drug administration has been emerged as a preferred method for managing gout, especially in localized inflammation sites, such as joints in the big toe (Dehlin et al., 2020, Tuszynski et al., 2021). It directly delivers the drugs into blood via non-invasive skin route with more sustaining and less frequent dosing. As compared to conventional delivery systems, the TDDS has vast benefits thus contributing a great interest for investigators (Farag et al., 2022). It has several notable advantages, such as the ability to achieve sustained and controlled drug release over an extended period, ensuring a consistent presence of the drug in the bloodstream. Additionally, this method enables the utilization of a passive delivery system through diffusion, further enhancing its benefits (Sabbagh & Kim, 2022). Several nanotechnology based formulations are in practice nowadays including the nanoemulsions, nanogels, nanospheres, nanocrystals, hydrogels films and micro-needles patches which are used for the transdermal delivery (Mohanto et al., 2023, Gowda et al., 2023).

Among these, nano-cubosomes (NCs) have emerged as lipid-based nano systems resembling established vesicular systems such as liposomes and niosomes. NCs present a unique opportunity to be incorporated into a novel drug delivery system, accommodating hydrophilic, lipophilic, and amphiphilic drug molecules (Umar et al., 2022). Nano-cubosomes comprising of paired systems GMO and water are the most extensively studied systems. These systems have the ability of self-assembling into liquid cubic crystalline as they have the characteristics of large interior surface area and viscid isotropic nature (Yosif et al., 2022). Their unique three dimensional (3-D) structures having lipophilic and hydrophilic subunits have made these nano-systems enable to be used as drug carrier (Tu et al., 2014). Due to the large interior surface area of the nano-cubosomes which provides these systems complex diffusion pathways, the encapsulated drug can be released in controlled pattern (Mohanto et al., 2023; Patra et al., 2018). Moreover the nano-cubosomes are biodegradable, bio-adhesive and biocompatible due to the presence of the lipid subunits (GMO). Following these features, the nano-cubosomes have been investigated and explored as versatile nanocarriers for the transdermal delivery of several therapeutic agents including peptides, enzymes, antibiotics and chemotherapeutics (Yosif et al., 2022).

Gout, the most prevalent form of inflammatory arthritis, is characterized by recurrent painful and swollen joint attacks. It arises from persistently elevated serum urate levels, a condition known as hyperuricemia (Berman et al., 2018). Hyperuricemia causes the accumulation of monosodium urate crystals (MSU) in various tissues, including joints. This leads to the occurrence of gout flares, which are characterized by acute inflammation and significantly impact the quality of life of affected individuals (Karantas et al., 2023 Aug 9, Dehlin et al., 2020). The global prevalence of gout has been on the rise, with a significant increase in affected individuals below the age of 30 (Choi et al., 2005). In 2017, the global estimation indicated a cumulative total of approximately 7.44 million cases of gout (with an incidence rate of 0.097 %, ranging from 0.086 % to 0.111 %) and a prevalence of around 41.22 million cases (with a prevalence rate of 0.54 %, ranging from 0.48 % to 0.60 %) (Roberts et al., 2019).

Capsaicin, the bio-active component found in chili has proven to be highly effective and widely utilized in topical preparations for treating gouty arthritis. It is classified as a BCS class II drug and often used as a topical painkiller and anti-inflammatory. However, when administered orally in humans, capsaicin undergoes significant hepatic first-pass metabolism, leading to gastrointestinal disturbances. Intravenous administration of CAP results in systemic side effects and exhibits a short half-life of approximately 7 min (Ostrovsky, 2017). Pain-sensing nerve endings are temporarily degraded by capsaicin, which works by stimulating neurons that are responsible for conveying pain signals to the brain. The inhibition of pain signal transmission by neurons provides long-lasting relief from pain. The significance of capsaicin extends to its well-established role in skeletomuscular disorders, prompting numerous studies exploring its anti-arthritic activity. The research conducted on capsaicin highlights its potential as a valuable therapeutic agent for alleviating arthritis-related symptoms and discomfort (Kumar et al., 2016). A study conducted by Barki et al (2020) incorporated capsaicin in dexibuprofen reinforced skin emulgel for transdermal delivery to synergize the anti-inflammatory and analgesic effects in animal models (Barki et al., 2020). Similarly another study conducted by Khan et al., (2021) prepared nanocrystals of capsaicin for alleviating pain and inflammation (Khan et al., 2021).

Thiocolchicoside (TCS) is a chemical of semi-synthetic nature that is obtained through the derivation of colchicoside. The drug functions by acting as an agonist for GABA receptors within the central nervous system (CNS), thereby producing anti-inflammatory, analgesic, and muscle-relaxant properties. Previously, the administration of this substance has been conducted through several routes, including oral ingestion, parenteral administration (IM), and topical application (Bigucci et al., 2015)-12382514016990. TCS is well-known for its muscle-relaxant, anti-inflammatory, and analgesic properties. It is a BCS class III drug that is frequently prescribed to the patients with orthopedic, rheumatologic, or musculoskeletal conditions to treat muscular pain and contractures (Bhamburkar et al., 2022). Paradkar et al (2018) reported a study on the preparation of TCS niosomal gel by thin film hydration method for pain management in rheumatoid arthritis. The study concluded that TCS based niosomal gel may prove as safe alternative to oral route and promising drug carrier for transdermal delivery of TCs for pain management in rheumatoid arthritis. The drug can be released in controlled manner thus increasing the retention time as well as the patient compliance with reduced dose frequency and associated side effects (Paradkar et al., 2018). TCS is an effective therapeutic agent indicated for rheumatologic disorders however it undergoes first pass metabolism leading to poor bioavailability and possess low permeability via oral route (Bhamburkar et al., 2022).

Therefore this study was aimed to formulate nanocubosomes loaded with capsaicin and thiocolchicoside to enhance their bioavailability and minimize associated potential side effects through transdermal delivery in nano-cubosomes (NCs) thus resulted in their synergistic activity.

## 2 Materials and methods

### 2.1 Reagents and chemicals used

Glycerol mono oleate (GMO) and Poloxamer 407 (P407) were purchased from SIGMA ALDRICH, USA. Capsaicin (CAP), and thiocolchicoside (TCS) were gifted from Standpharm Pvt limited Lahore, Pakistan. Ethanol 95 % (HPLC grade; Sigma Aldrich), Distilled water, and deionized water (Institute of Chemical Sciences, Gomal University Dera Ismael Khan, Pakistan), Polyethylene glycol (PEG 400), Triethanolamine, and Tween-80 (Sigma Aldrich, St. Louis, MO, USA) were used. Voltral® EmulGel 1 % (Novartis Pvt. Ltd, Karachi), Phosphate buffer and Acetate buffer solutions were also used. All the chemicals and reagents used in this study were of analytical grades.

### 2.2 Experimental design

DESIGN EXPERT® software, version 12, by Stat-Ease Inc., Minneapolis, MN, USA, was used to conduct experiments using a Box-Behnken design. The study focused on investigating the impact of three independent variables: concentrations of CAP and TCS (drugs), GMO (lipid), and P407 (stabilizer) at three levels i.e. low, medium and high (Table 1).

**Table 1:** Box-Behnken factorial and its dependent as well as independent variables.

**Independent variables**	Levels		
	**Low**	**Medium**	**High**
GMO amount (lipid) g	3.035	4.884	6.070
P 407 amount (stabilizer) g	0.996	0.814	0.632
CAP concentration (Active drug) mg	0	2.5	5
TCS concentration (Active drug) mg	0	2.5	5

**Dependent variables**	**Units**	**Goals**	
Mean particle size	nm	Minimize	
EE	Percent	Maximize	
Zeta potential	mV	Maximize	
PDI	---	Minimize	

### 2.3 Preparation of blank NCs

The NCs were prepared by modified emulsification method as reported by Yosif et al (2022), slightly revised (yosif et al., 2022). For this purpose, precise amounts of glycerol mono oleate (GMO), poloxamer 407 (P407), and water were weighed accordingly. Initially, GMO and P407 were heated at 70 ± 5 °C in a hot water bath for 30 min. While constantly stirring at 1000 rpm, the molten solution was added to deionized water at 70 °C containing the emulsifier (PEG 400) for optimum homogeneity. In order to get a uniform combination, the resultant dispersions were stirred continually for an additional two hours before being allowed to equilibrate at room temperature (25 ± 2 °C). A high speed homogenizer (Euro-Star, IKA D 230, Germany) was used at 12000 rpm for 10 min to achieve a uniform dispersion in the NCs throughout the homogenization process. The NC dispersion was then subjected to a two-minute period of vortex mixing, followed by five minutes of probe sonication. The lipid droplets were allowed to solidify at room temperature.

### 2.4 Preparation of CAP & TCS co-loaded nanocubosomes (CAP/TCS NCs)

To prepare drug-loaded NCs, varying concentrations of capsaicin was added to the oily phase. Similarly varying concentration of thiocolchicoside was added to the aqueous phase before mixing. The subsequent steps for the preparation of drug-loaded NCs were identical to those described for the preparation of blank NCs. The resulting milky NC dispersions were stored at room temperature for further analysis. The composition of NCs has been given in Table 2.

**Table 2:** Composition of blank and drugs loaded nano-cubosomes.

**F. Code**	**A:CAP** **mg**	**B:TCS** **mg**	**C:P407** **g**	**D:GMO** **g**	**QS to make 25 mL**
1	2.5	5	0.814	3.035	25
2	0	2.5	0.632	4.884	25
3	2.5	0	0.814	3.035	25
4	2.5	5	0.814	6.070	25
5	2.5	2.5	0.632	3.035	25
6	2.5	0	0.632	4.884	25
7	2.5	5	0.996	4.884	25
8	5	0	0.814	4.884	25
9	0	2.5	0.814	3.035	25
10	0	0	0.814	4.884	25
11	5	2.5	0.814	6.070	25
12	2.5	2.5	0.996	6.070	25
13	0	5	0.814	4.884	**25**
14	2.5	2.5	0.996	3.035	25
15	2.5	2.5	0.814	4.884	25
16	5	2.5	0.632	4.884	25
17	5	5	0.814	4.884	25
18	5	2.50	0.814	3.035	25
19	2.5	2.5	0.632	4.884	25
20	2.5	2.5	0.814	4.884	25
21	0	2.5	0.814	6.070	25
22	2.5	2.5	0.814	4.884	25
23	0	2.5	0.996	4.884	25
24	2.5	5	0.632	4.884	25
25	2.5	0	0.814	6.070	25
26	5	2.5	0.996	4.884	25
27	2.5	0	0.996	4.884	25

### 2.5 Physicochemical characterizations

#### 2.5.1 Homogeneity test

All NC formulations were visually examined for factors like colour, odour, uniformity grittiness, consistency, and phase separation (Burki et al., 2020).

#### 2.5.2 Dilution test

The physical stability of NC dispersion was checked with respect to dilution by carrying out dilution test. Briefly, 1 mL of the formulation was diluted to 50 mL, 100 mL and 500 mL with the addition of double distilled water with constant stirring at different speed of 50 rpm, 500 rpm and 1000 rpm. Phase separation, cracking, turbidity and clarity were observed (Sabbagh & Kim, 2022).

#### 2.5.3 Separation test

NC dispersions were checked for physical stability by performing separation test. Phase separation was checked and evaluated by centrifugation for 30 min at 5000 rpm (Badri et al., 2014).

#### 2.5.4 Spreadability test

The spreadability of optimized NC dispersion is very important and was analyzed by the “Drag & Slip” method, as described by Dhadwal et al (2020), with minor modifications (Dhadwal et al., 2020). This tool is made of wood-block with 2 glass slides (one stationary block and other mobile), a pulley is attached to one terminal. This test is crucial to evaluate the ability of semisolid dosage forms to spread on the skin with minimal shear application. It plays a significant role in ensuring the effective administration of a standardized dose of the medicated formulation onto the skin. A 2 g sample of the optimized NC dispersion was placed between two glass slides and subjected to a 50 g weight for 5 min. The time (seconds) taken by the upper slide to move was noted by the following equation,

(1) S=(M×L)/T

Where **S** represents spreadability of the NC dispersion, **M** is the mass attached to the top/upper slide, **L** is the length of the slides and **T** is the time it takes to move.

#### 2.5.5 Viscosity test

It controls the NCs' drug release rate, affecting the formulation's effectiveness and therapeutic potential. The viscosity of NC formulation was determined at various temperature, i.e., 8 °C, 25 °C and 40 °C by using Brookfield viscometer with spindle number 3 and the viscosity was recorded at the predetermined time periods 0 min, on day 1, after 2 days, 7, 14 and 28 days, and was measured in cps at different rpm i.e. 6, 12, 24, 30 (Mehta et al., 2021).

#### 2.5.6 Conductivity test

The conductivity test is essential for characterizing the nature of the NCs formulations. The electrical conductivity is measured and logged in s/cm by putting the conductometer probe within 10 mL of the mixture at room temperature. This test helps to distinguish between water-in-oil (w/o) and oil –in-water (o/w) emulsions. Formulations showing no conduction due to the internal water phase are w/o emulsions, while those exhibiting high conduction as a result of the continuous external aqueous phase are o/w emulsion (Al-sakini & Maraie, 2019b).

#### 2.5.7 Stability studies

Optimized formulations of NCs co-loaded with capsaicin and thiocolchicoside were subjected to different temperatures and humidity conditions for stability testing. The formulations were tested for 28 days in a series of stability experiments that evaluated their stability at relative humidity of 75 % and three distinct temperatures, low temperature (8 °C), at room temperature of 25 °C, and increased temperature of 40 °C (Yosif et al., 2022).

#### 2.5.8 Determination of pH

The pH of the NCs dispersions was measured using a digital pH meter (Denver, USA). For each NCs sample, the pH sensor probe electrode was carefully immersed, and the system was allowed to stabilize for 2 min (n = 6). The pH of the NC dispersion was then recorded from the digital display. The pH measurement was repeated at predetermined time intervals, at 0 min, 12, 24, 36, 48, 72 h, 1st week, after 2, 3, and 4 weeks. All of the readings were taken three times to ensure accuracy (Burki et al., 2020).

#### 2.5.9 Zeta analysis

Using a Zetasizer (Malvern Instruments, UK) at 25 ± 0.5 °C, the particle/droplet size distribution (mean diameter), surface charge (zeta potential), and polydispersity index were measured. Prior to measurements, the samples were appropriately diluted (50 folds) with deionized water to adjust the signal level. The average results were computed after three independent samples were measured three times each (Khan et al., 2021).

#### 2.5.10 FTIR analysis

NCs were characterized using Fourier Transform Infrared Spectroscopy (FTIR) analysis technique to study functional groups and intramolecular interactions between the drug molecules and formulation’s ingredients. The FTIR spectroscopy was performed using a Bruker instrument (Karlsruhe, Germany) following the method described in literature (Khan et al., 2021). In summary, only a small amount of CAP, TCS, CTNCs, and blank NCs were placed immediately in the sample chamber. The IR spectra were analyzed in range of 500 to 4000 cm − 1, its scanning speed was kept at 2 mm sec − 1 for 120 scans and at a resolution of 4 cm − 1.

#### 2.5.11 Transmission electron microscopy (TEM)

The morphology of optimized NC co-loaded with CAP and TCS was visualized by using TEM (TEM; JEOL JEM-1010, Tokyo, Japan). For this purpose, the sample was diluted and one drop of diluted sample was placed on a carbon-coated copper grid. Next, they were stained with 2 % (w/v) phosphotungistic acid (negative staining technique). The sample was visualized using TEM with magnification power of X80000 after drying at room temperature and an accelerated voltage of 80 KV (Bhosale et al., 2013).

#### 2.5.12 Entrapment efficiency (E.E) and drug loading (DL)

Entrapment efficiency (E.E) gives us an idea about the percent drug (% drug) that is entrapped or absorbed successfully into the nano-composite and drug loading capacity helps to deal with the nanocompites after their separation from the medium and find out their drug content (Yue et al., 2009). To separate free CAP and TCS from the NC dispersion, Ultrafiltration centrifugation technique was used, allowing for the measurement of drug loading (DL %) and entrapment efficiency (EE %) (Chung et al., 2002). The addition of 9.0 mL ethanol to a 1 mL nano-cubosomal dispersion containing the full amount of CAP and TCS was used to determine the magnitude of entrapment for both drugs. Using ethanol as a blank, a UV spectrophotometer was used to conclude the total amount of CAP and TCS present in the resulting solution. In order to dilute one mL solution of freshly synthesized NCs (co-loaded with CAP and TCS), 10 mL of distilled water was used. After dilution, 3 mL of the samples were centrifuged at 4000 rpm for 15 min. The concentration of free CAP and TCS un-entrapped in the supernatant was calculated using UV spectrophotometry at 280 nm and 259 nm, respectively (Pharmacopoeia & Commission, 2009). Encapsulated CAP and TCS concentration in 1 mL of NC dispersion was determined by subtracting the observed concentration of CAP and TCS in the filtrate from the total drug concentration.

Results were calculated in percentage using the following equations;

(2) $$\mathrm{DL}\%=\frac{\mathrm{Conc}.\;of\;CAP/TCS\;in\;NCs\neg \neg \neg \neg \times 100}{\mathrm{Weight}\;of\;NCs}$$

(3) $$\mathrm{EE}\%=\left(C_{\mathrm{totalconc}}-C_{\mathrm{freeconc}}\right)/C_{\mathrm{totalcon}}\times 100$$

### 2.6 In vitro drug release

The *in vitro* drug study was conducted as per reported study of Khan et al., slightly modified using unjacketed vertical Franz Diffusion Cells, which had a diffusional surface area of 5.96 cm^2^ and a receptor cell volume of 20 mL (Khan et al., 2021). The donor compartment contained a formulation equivalent to 2.5 mg of capsaicin and 5 mg of thiocolchicoside. Phosphate buffer (PB) pH 7.4, was used to fill the receptor compartment; 0.02 % w/v ethanol was added to the PB to prevent microbial growth; and the entire process was retained at a temperature of 37 ± 2 °C with continuous stirring for 24 h. The donor chamber and the sample port were both covered with lids to prevent evaporation during the trial. Samples were taken using a spinal syringe every 0, 5, 10, 15, 30, 60 min, 2, 3, 4, 5, 6, 7, 8, 12, and 24 h. Drug concentrations were determined using ultraviolet (UV) spectrophotometry after the samples had been properly diluted at the end of the study (Gaballa et al., 2020).

### 2.7 Ex-vivo studies

The *ex vivo* permeability of NCs co-loaded with CAP and TCC has been tested using Franz diffusion cells. A freshly excised patch of hairless abdominal rat skin was clamped between the donor & receptors sections, with the stratum corneum towards the donor compartment to increase the effective permeation area to 2 cm^2^. At a steady rate of 60 rotations per minute, a magnetic bar was used to stir the receptor solution while maintaining a temperature of 37 ± 0.5 ͦ C. The donor reservoir was filled with a 1 mL dispersion of CAP/TCS in normal saline. In 1 mL samples obtained at regular intervals from the receptor compartment, the drug content of CAP and TCC UV was determined with a UV spectrophotometer at 280 nm and 259 nm, respectively. In this experiment, we used a phosphate buffer solution with a pH of 5.4, which was maintained at a temperature of 37 ± 0.5 °C (Chettupalli et al., 2021).

### 2.8 Skin retention

The skin retention of NCs was determined following a method described in the literature with slight modifications (Baveloni et al., 2021). The NCs were left on the skin for 24 h before being rinsed off with distilled water and the remaining formulation was cleaned with cotton swabs. After that, 15 pieces of sticky tape was used to remove the stratum corneum of the skin, and then the tapes holding the stratum corneum were soaked in 5 mL of methanol. After being probe sonicated for 10 min, the tapes were subjected to vortexes for two minutes each. After removing the epidermis and dermis, we chopped it into tiny pieces, dissolved it in methanol and sonicated it for 10 min. The capsaicin and thiocolchicoside contents of all samples were determined by centrifuging them at 15,000 rpm for 10 min and then collecting the supernatant for UV spectrophotometer analysis.

### 2.9 In vivo study

#### 2.9.1 Ethi cal approval

With reference number 456/ERB/GU (dated: 21–06-2023), the Institutional Ethical Review Board of Gomal University. D.I. Khan, Pakistan, approved the *in vivo* investigation using animal models. All animals used in experiments were treated in compliance with the recommendations laid forth by the National Institutes of Health's Guide for the Care and Use of Animals in Laboratory Work.

#### 2.9.2 Experimental animals

Studies on anti-inflammatory and analgesic efficacy were conducted in male Wistar Albino rats (*Rattus norvegicus*) having 180–220 g of body weight and in male Swiss Albino mice (*Mus musculus*) with body weight of 25–35 g. Standard laboratory conditions were maintained for the animals, including a humidity range of 65–70 %, a room temperature of 22 ± 2 °C, and light/dark cycle of 12-hours each. Before the *in vivo* research started, they spent seven days getting used to the lab environment and closely monitored their food and drink consumption.

#### 2.9.3 Assessment of skin irritation

To assess potential skin irritation caused by the different NC formulations co-loaded with capsaicin and thiocolchicoside, a skin irritation study was conducted in mice. Application of the capsaicin and thiocolchicoside-containing NC formulation was carried out twice per day for 24 h on a small (22 cm^2^) area of the mouse's back skin to check any signs of redness, erythema, edema and allergic reactions (Rai et al., 2018).

#### 2.9.4 Carrageenan induced rat paw edema assay

Study on the anti-inflammatory activity was conducted in Wistar albino rats, which were 180–220 g in weight. Each rat's right hind paw was interplanetary injected with 0.1 mL of 1 % w/v carrageenan solution (freshly prepared) in normal saline to cause acute inflammation (paw edema), as per the guidelines given by Badole et al, (2012). Carrageenan-induced paw edema served as the basis for evaluating the anti-inflammatory effect. The anti-inflammatory activity was examined after application of treatment with plain NC base, standard diclofenac diethylamine (Voltral® EmulGel) 1 % and test CAP-TCS NC dispersion. After administering the treatments, the index finger was gently massaged 50 times over the surface of the hind paw. The control group of rats was given treatment of only plain NC dispersion base. Diclofenac diethylamine 1 % was applied to standard group of rats. The formulated NCs co-loaded with CAP and TCS was applied to test group of rats. The treatments with plain base, standard drug and test NC drug loaded dispersion were applied 30 min before injecting carrageenan. The paw thickness (mm) was instantly measured at 0, 30, 1, 2, 3, 4 h after administration of the treatments by using digital Vernier caliper (Vasudevan et al., 2007).

The % inflammation inhibition was calculated as follows:

(4) %inflammationinhibition=(CG-T/CG)×100

CG = Carrageenan Group

T = Test Formulation

#### 2.9.5 Analgesic activity by hot plate method

The male Swiss Albino mice (*Mus musculus*) were used as experimental animals for analgesic activity. The analgesic activity was evaluated by using Eddy’s hot plate method (Eddy and Eimbach, 1953). The mice were divided into 3 groups. Group I designated as control group received treatment of plain nano-cubosomal dispersion. Group II assigned as standard group received treatment of transdermal diclofenac diethylamine 1 % (Voltral^±^ EmulGel). Group III served as test group received transdermal treatment of nano-cubosomes co-loaded with capsaicin and thiocolchicoside. The experimental animal was placed separately on hot plate maintained at temperature 55 ± 2C. The reaction time (licking time) was noted for each mice at different time intervals of 0 min, 30 min, 60 min and 90 min after application of different treatments with cut off time 15 sec to avoid tissue damage (Burki et al., 2020).

### 2.10 Statistical analysis

The results of the experiments were reported in the form of the mean ± Sd. DESIGN EXPERT® software, (version 12, by Stat-Ease Inc., Minneapolis, MN, USA) was used for the statistical analysis. Results with *P ≤ 0.05* were taken significant while those with *P ≥ 0.05* were insignificant.

## 3 Results

### 3.1 Optimization of NCs co-loaded with capsaicin and thiocolchicoside

To optimize the nano-cubosomes (NCs), a Box-Behnken experimental design (3^2^ factorial designs) was employed using Stat-Ease Inc.'s DESIGN EXPERT® software, version 12, based in Minneapolis, MN, USA. The impact of three key variables concentrations of CAP, TCS drugs, GMO polymeric lipid, and P407 stabilizing agent at three different levels (low, medium and high) were focused. These variables were investigated for their influence on important characteristics of the NCs, including particle/droplet size (measured in nm), entrapment efficiency (expressed as a percentage), PDI and zeta potential (measured in mV).

### 3.2 Selection of optimized formula

**Table:** 

**Independent variables**	**Levels**		
**Low**	**Medium**	**High**	
GMO amount (lipid) g	3.035	4.884	6.070
P 407 amount (stabilizer) g	0.996	0.814	0.632
CAP concentration (Active drug) mg	0	2.5	5
TCS concentration (Active drug) mg	0	2.5	5
**Dependent variables**	**Units**		**Goal**
Mean particle size	nm		Minimize
EE	Percent		Maximize
Zeta potential	mv		Maximize
PDI	---		Minimize

**Fig. 1:**
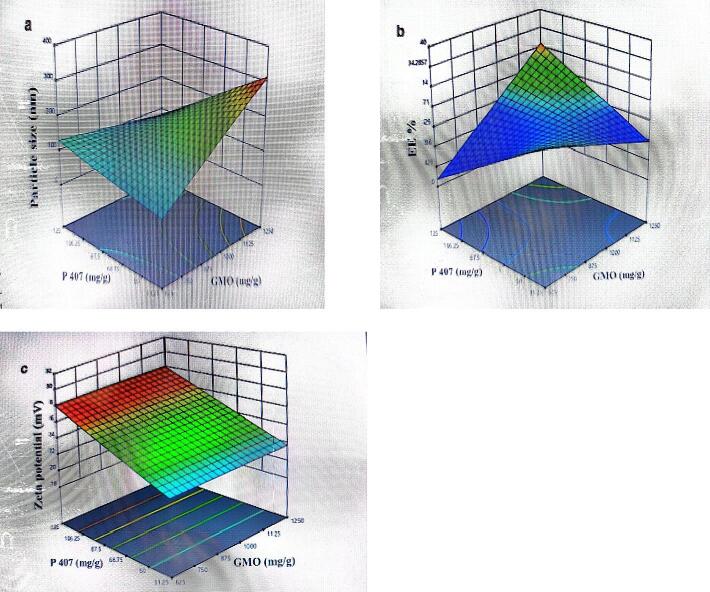
Effects of independent variables on (a) particle size distribution (b) entrapment efficiency (%), and (c) zeta potential.

Zeta potential, particle size, polydispersity index (PDI), and entrapment efficiency were found to be significantly correlated with the concentrations of GMO, P407 and drugs (both CAP & TCS) in NC formulations. Particle size, E.E %, and zeta potential were evaluated as functions of the relationship between P407 and GMO quantities using three-dimensional surface diagrams. Particle size was shown to be directly proportional to the GMO/P407 ratio **(**Fig. 1**,** Table 3**)**.

**Table 3:** Tests conducted using a Box-Behnken experimental layout.

F. Code		Values of Independent Variables (Factor)				Values of Dependent Variables (Response)			
Std	**Run**	**A:CAP** **(mg)**	**B:TCS** **(mg)**	**C:P407** **(g)**	**D:GMO** **(g)**	**Particle size** **nm**	**PDI**	**Entrapment efficiency** **%**	**Zeta potential** **mV**
22	1	2.5	5	0.814	3.035	514	0.536	76.20	−8.43
17	2	0	2.5	0.632	4.884	510	0.532	82.2	−8.69
21	3	2.5	0	0.814	3.035	502	0.528	82.9	−8.78
24	4	2.5	5	0.814	6.070	520	0.405	84.3	−10.0
5	5	2.5	2.5	0.632	3.035	509	0.532	79.1	−8.59
13	6	2.5	0	0.632	4.884	512	0.562	79.6	−8.42
16	7	2.5	5	0.996	4.884	517	0.560	80.3	−8.69
2	8	5	0	0.814	4.884	501	0.405	82.4	−8.72
9	9	0	2.5	0.814	3.035	492	0.556	78.2	−7.97
1	10	0	0	0.814	4.884	220	0.405	00	−2.47
12	11	5	2.5	0.814	6.070	518	0.408	84.6	−8.69
8	12	2.5	2.5	0.996	6.070	516	0.542	81.1	−8.61
3	13	0	5	0.814	4.884	502	0.532	79.3	−8.43
6	14	2.5	2.5	0.996	3.035	490	0.571	80.0	−8.98
25	15	2.5	2.5	0.814	4.884	512	0.534	84.8	−8.70
18	16	5	2.5	0.632	4.884	519	0.406	85.9	−8.69
4	17	5	5	0.814	4.884	520	0.405	86.4	−10.0
10	18	5	2.50	0.814	3.035	502	0.536	81.5	−8.42
7	19	2.5	2.5	0.632	4.884	518	0.407	83.6	−8.68
27	20	2.5	2.5	0.814	4.884	514	0.538	81.8	−8.44
11	21	0	2.5	0.814	6.070	501	0.567	80.8	−8.44
26	22	2.5	2.5	0.814	4.884	500	0.534	80.4	−8.79
19	23	0	2.5	0.996	4.884	496	0.562	81.3	−8.73
14	24	2.5	5	0.632	4.884	503	0.405	86.9	−10.0
23	25	2.5	0	0.814	6.070	520	0.536	81.1	−8.64
20	26	5	2.5	0.996	4.884	510	0.544	82.0	−8.52
15	27	2.5	0	0.996	4.884	498	0.556	81.9	−8.78

*F10: Optimized Blank NCs.

*F24: Optimized Drug loaded NCs.

### 3.3 Effect of formulation variable (Glyceryl monooleate; GMO) amount on the average particle size and entrapment effectiveness of developed NCs

With the increase in GMO amount, there was an observed increase in both the average particle size from 206 to 503 nm and the drug entrapment efficiency from 76.20 % to 86.9 %. The optimal formulation of nano-cubosomes (NCs) was achieved by employing an optimal concentration of GMOs at 4.5 %. This led to an average particle size of 503 nm and an entrapment efficiency of approximately 86.9 %.

### 3.4 Effect of formulation variable (poloxamer 407) amount on mean particle size and entrapment efficiency of developed nano-cubosomes

It was observed that ideal poloxamer 407 concentration of 0.5 % yielded optimized nano-cubosomal formulations thus showed average particle size of 503 nm and entrapment efficiency about 86.9 %.

### 3.5 Homogeneity test

It was observed that the prepared NC formulations appeared as white homogenous milky dispersion with no visible signs of aggregates, grittiness and lumps.

### 3.6 Dilution test

The aforementioned test was of significant importance in elucidating the characteristics of the NC dispersion in distinguishing between water-in-oil (w/o) and oil-in-water (o/w) configurations. For o/w dispersions, no cracking or phase separation was observed even with the addition of more continuous phase (water). All the NC dispersions (F1-F27) demonstrated clear and stable dispersion without any observable cracking or phase separation in less than 1 min, confirming that all the dispersions were of o/w nature.Table 4.

**Table 4:** Physical stability of blank and drugs loaded NCs.

**Formulation**	**Physical Stability**			
	**Phase Separation**	**Cracking**	**Turbidity**	**Clarity**
F10- BNCs	Not seen	Not seen	Not seen	Passed
F24- CTNCs	Not seen	Not seen	Not seen	Passed

**F10- BNCs**: Optimized blank NCs.

**F24- CTNCs**: Optimized drugs loaded NCs.

### 3.7 Separation test

Upon subjecting the prepared NC formulations (F1-F27) to centrifugation, it was noticed that no phase separation, creaming, or sedimentation happened, indicating a high level of stability in these formulations. The stability of the formulations may have been aided by the dispersion droplets' thermal motion, or Brownian motion, surpassing external forces like gravitational pull and centrifugation.

### 3.8 Spreadability studies

The spreadability of semi-solid dosage forms is assessed by evaluating their capacity to disperse over the skin with the application of minimal shear force. As the poloxamer 407 concentration increased from 0.6 % to 2.4 % (F1-F27), the NCs' viscosity also increased. As a result, the observed increase in viscosity resulted in a decrease in spreadability, which is from 3.3 cm to 2.5 cm. The study determined that the spreadability of optimal dispersion was measured to be 11.32 gm.cm/sec. Data is shown in Table 5.

**Table 5:** Average spreadability values of blank and drug co-loaded nano-cubosomes kept at various temperatures i.e; 8 °C, 25 °C and 40 °C.

**Formulation**	**Spreadability (****g.cm/sec****)**		
	**Temperatures**		
	**8 °C**	**25 °C**	**40 °C**
**F10**	10.98	11.75	13.42
**F24**	8.84	9.70	11.32

**F10:** Blank NCs.

**F24:** CAP/TCS co-loaded NCs.

### 3.9 Viscosity studies

The optimized NC dispersion F24 exhibited a viscosity ranging from 2378 to 8234 cP as shown in Table 6, Table 7 at different indicated temperatures.

**Table 6:** Average viscosity values of blank nano-cubosomes kept at 8 °C, 25 °C and 40 °C at various time intervals.

**Time Period (Days)**	**Viscosity (centipoise)**					
	**8 °C**		**25 °C**		**40 °C**	
	**6 rpm**	**12 rpm**	**6 rpm**	**12 rpm**	**6 rpm**	**12 rpm**
**0**	8238	4208	8238	4208	8238	4208
**7**	8235	4201	8222	4176	8167	4180
**14**	8232	4196	8209	4162	8051	4166
**21**	8228	4189	8198	4141	7898	4142
**28**	8221	4179	8185	4132	7706	4121

**Table 7:** Average viscosity values of F24 optimized nano-cubosomal dispersion kept at 8 °C, 25 °C and 40 °C at various time intervals.

**Time Period (Days)**	**Viscosity (****m.Pa****.s)**					
	**8 °C**		**25 °C**		**40 °C**	
	**6 rpm**	**12 rpm**	**6 rpm**	**12 rpm**	**6 rpm**	**12 rpm**
**0**	3596	2623	3596	2623	3596	2623
**7**	3586	2642	3581	2612	3578	2606
**14**	3574	2630	3561	2593	3558	2580
**21**	3546	2614	3542	2569	3544	2554
**28**	3533	2594	3512	2555	3532	2412

### 3.10 Conductivity test

Conductivity test is a crucial method to determine the nature of NC dispersions and identify phase inversion. In our study, the higher conductivity values (ranging from 0.0897 to 0.157 ms/cm) specified that all NC dispersions were o/w type, where water served as the external phase.

### 3.11 pH determination

The pH values of the nano-cubosomal formulations (F1-F27) were determined in triplicate using a digital pH meter, yielding a range of 5.56 to 6.28 (Table 8).

**Table 8:** pH of blank and drugs co-loaded nano-cubosomes.

**Time Period (Days)**	**pH**					
	**Temperatures**					
	**8 °C**	**8 °C**	**25 °C**	**25 °C**	**40 °C**	**40 °C**
	**BNCs**	**CTNCs**	**BNCs**	**CTNCs**	**BNCs**	**CTNCs**
**0**	6.28	5.92	6.28	5.86	6.28	5.72
**7**	6.23	5.85	6. 17	5.82	6.14	5.59
**14**	6.17	5.73	6.14	5.67	6.09	5.36
**21**	6.09	5.56	6.03	5.51	5.97	5.20
**28**	5.99	5.41	5.79	5.14	5.46	4.98

*BNCs: Blank nano-cubosomes.

*CTNCs: Capsaicin-Thiocolchicoside co-loaded nano-cubosomes.

### 3.12 Particle/droplet size, zeta potential, and polydispersity index (PDI)

On average, droplet particulate size was between 206 and 503 nm confirming that the prepared NC dispersions had an effective diameter within the nanometer size range. The observed PDI ranged from 0.405 to 0.440. The zeta potential values of NCs dispersions (F1-F27) ranged from −2.47 mV to −10.0 mV. The data has been presented in Table 9.

**Table 9:** Evaluation of the particulate size, PDI, and zeta potential of both blank nano-cubosomes and CAP-TCS NCs.

**Formulation Code**	**Average Particle Size (nm)**	**Zeta** **Potential (mV)**	**PDI**
**F10 (Blank Nano-cubosomes)**	206.0 ± 3.90	−2.47 ± 0.32	0.405 ± 0.02
**F24 (Drugs co-loaded Nano-cubosomes)**	503.0 ± 10.30	−10.0 ± 1.70	0.440 ± 0.03

### 3.13 FTIR analysis

**Fig. 2:**
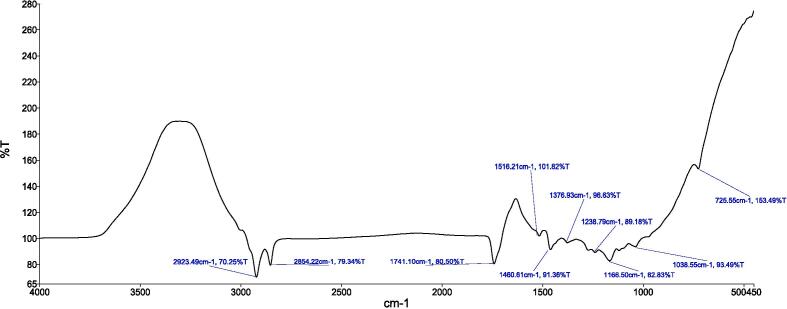
FTIR spectrum of pure capsaicin.

**Fig. 3:**
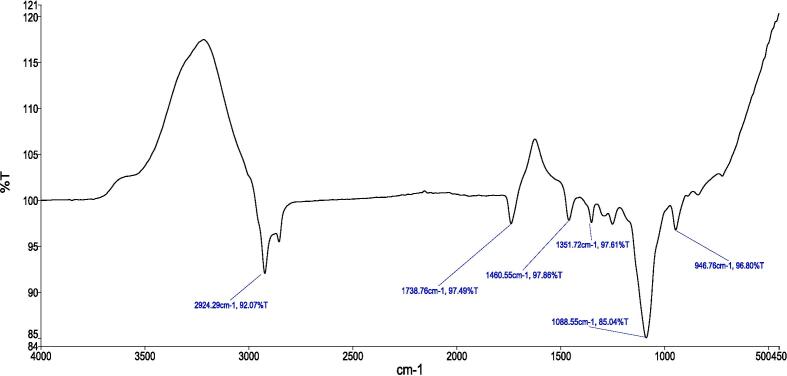
FTIR spectrum of pure TCS.

**Fig. 4:**
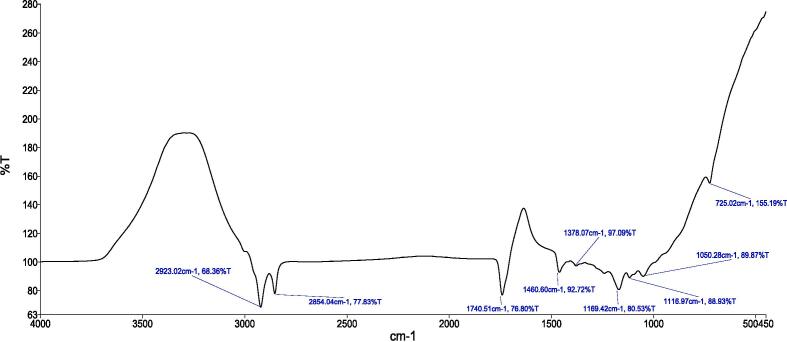
FTIR spectrum of lipid GMO based BNCs.

**Fig. 5:**
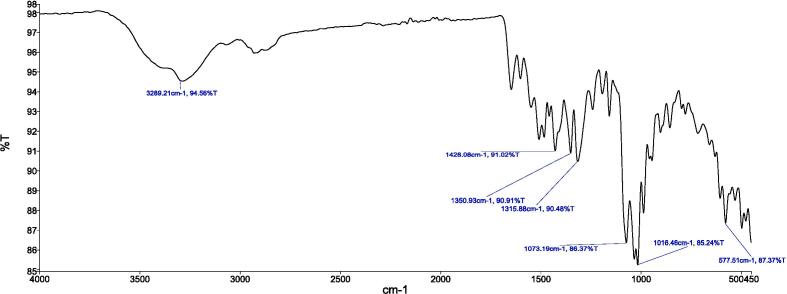
FTIR Spectrum of CTNCs.

Fig. 2, Fig. 3, Fig. 4, Fig. 5 provide a visual representation of the FTIR spectra for distinct components: pure capsaicin (CAP), pure thiocolchicoside (TCS), lipid GMO-based NCs, and the capsaicin-thiocolchicoside co-loaded nano-cubosomes (CTNCs). The FTIR spectrum of pure capsaicin illustrates distinct peaks at specific wavenumbers, such as 2923.49 cm − 1 (related to C–O–C vibrations), 2854.22 cm − 1 (C–O vibrations), 1741.10 cm − 1 (C–H bending vibrations), 1460.61 cm − 1 (C = C and C = O vibrations), 1166.50 cm − 1 (indicating stretching vibrations of the benzene ring), 1038.55 cm − 1 (C = C stretching representing an aromatic moiety), and 725.55 cm − 1 (–OH vibrations). Meanwhile, the FTIR spectrum of thiocolchicoside (TCS) showcases significant peaks at distinctive points including 2924.29 cm − 1 (N–H stretching vibration), 1738.76 cm − 1 (C–H stretching vibration), 1460.55 cm − 1 (C = O stretching vibration), 1351.72 cm − 1 (C = C stretching vibration), and 1088.55 cm − 1 (C-N stretching vibration). Also, the FTIR analysis of BNCs portrays discernible characteristic peaks for GMO at 2923.02 cm − 1 and 2854.04 cm − 1, along with F127 peaks at 1740.51 cm − 1 (pertaining to C–H stretching), 1460.60 cm − 1, 1169.42 cm − 1, 1116.97 cm − 1, and 1050.28 cm − 1.

### 3.14 TEM analysis

**Fig. 6:**
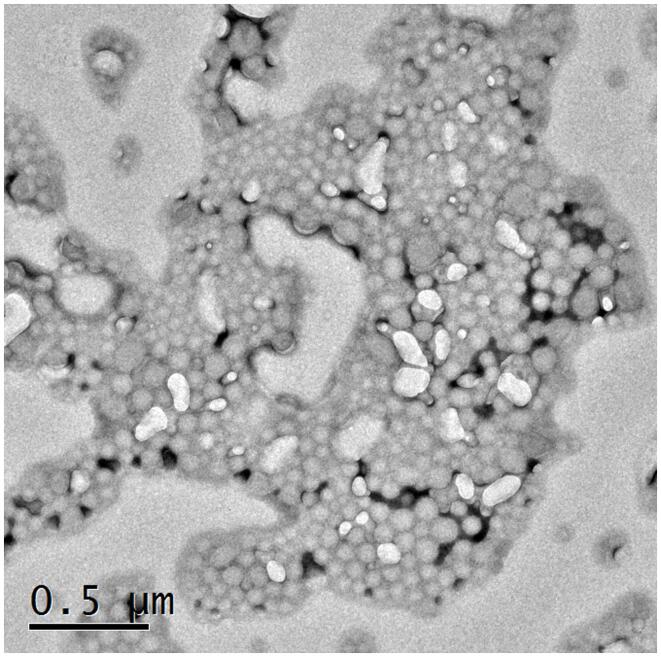
Morphological evaluation of NC by TEM.

TEM analysis was employed to visualize the morphology of optimized NCs as shown in Fig. 6. The TEM micrograph indicated that the prepared nano-cubosomes (NCs) are cubic in shape, uniform in size and well dispersed with no aggregates. The TEM micrograph revealed that the prepared nano-cubosomes (NCs) are in the nanosize which confirms that their particle size was compatible with the results of the particle size measurement as obtained by the Zetasizer.

### 3.15 Entrapment efficiency

It was found that the range of entrapment efficiency (%) for the NC formulations was approximately between 76.20 ± 2.90 % and 86.9 ± 3.6 5 %.

### 3.16 In vitro drug release

Using a Franz Diffusion Cell and a cellulose acetate membrane, the *in vitro* drug release profile of the optimal NC dispersion was evaluated in phosphate buffer (pH 7.4). The cumulative percentage of both Cap and TCS released from the NC dispersion over 24 h was 86.28 ± 6.66 % and 88.54 ± 6.87 %, respectively. A remarkable R2 value of 0.835 was found between the *in vitro* drug release kinetics of optimal NC dispersion and the Korsmeyer Peppas model.

### 3.17 Skin retention

The skin retention was observed to be higher in formulation (F24). When compared to the pure drug, F24 showed up to a six fold increase in skin retention.

### 3.18 Ex-vivo permeation

The *ex-vivo* evaluation of drug penetration was conducted on the dispersion of optimal NCs dispersion using rat skin as the substrate. In the current study, the evaluation of drug permeation was accomplished through a Franz diffusion cell. The cumulative percentage of both Cap and TCS permeated through skin for 24 h was 77 % and 84.54 %, respectively.

### 3.19 Skin irritation study

The safety of topical formulations is confirmed by skin irritation test. These formulations must not cause any contact dermatitis, irritation and allergy after applied to the skin. The skin area of the mice were checked for the presence of edema, redness and erythema. No edema, redness and erythema were found on the mice skin, which confirmed the suitability of prepared NCs for topical application.

### 3.20 Anti-inflammatory activity

**Fig. 7:**
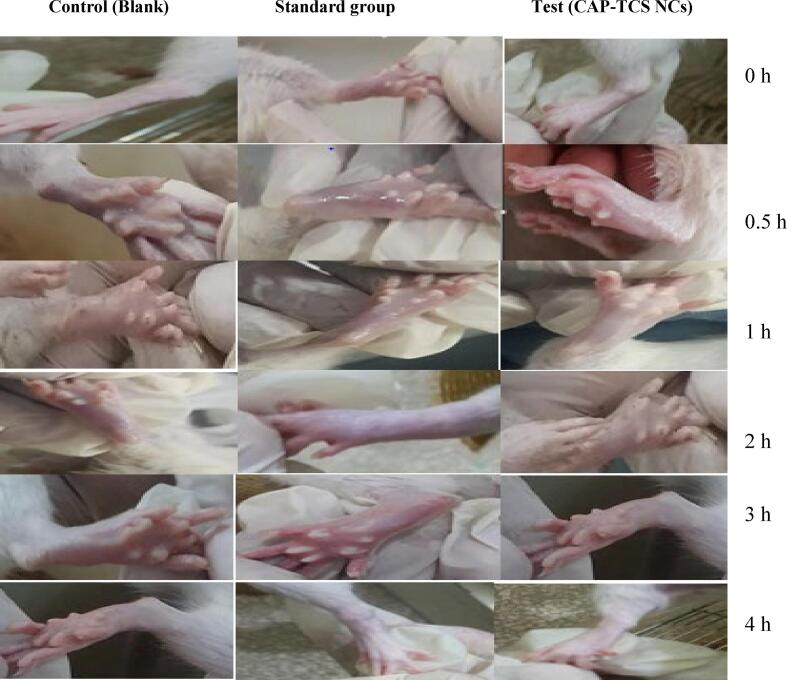
Photographic evaluation of paw edema inhibition in control, standard and experimental group animals.

Fig. 7 illustrates the final results of this activity. The test formulation reduced the volume of paw edema in a dose-dependent manner. Thirty minutes after injecting carrageenan into the rat's paw's sub-plantar region, an inflammatory response (edema) is visible. The biphasic nature of the presumed mechanism responsible for carrageenan-induced edema is widely acknowledged. The initial phase, which typically lasts for duration of 1 to 2 h, is associated with the release of serotonin, histamine, and bradykinin. On the other hand, the subsequent phase is characterized by the release of prostaglandins. After 3 h, the experimental formulation showed a significant decrease in paw volume, which suggests that the newly created NCs containing both capsaicin and thiocolchicoside may be able to suppress the release of prostaglandins during the second phase. After 3 h, the test formulation significantly (p < 0.001) decreased the volume of paw edema in contrast to the control group. The findings shown in Table 10 indicate that diclofenac, a widely recognized standard reference, exhibited a significant level of inhibition in lowering the volume of paw edema after a duration of 3 h (p < 0.001).

**Table 10:** Anti-inflammatory activity of standard, test formulation and blank formulation in experimental animals.

**Change in Hind Paw Edema Volume (mm) Mean ± SEM**						
**Treatment Groups**	**0 h**	**0.5 h**	**1 h**	**2 h**	**3 h**	**4 h**
**Standard Formulation (Voltral^±^ EmulGel 1 %)**	3 mm	4.5 mm	4.7 mm	4.2 mm	4.0 mm	3.8 mm
**Test Formulation (Drugs Co-loaded Nano-cubosomes)**	3.5 mm	5.5 mm	6.8 mm	3.5 mm	3.2 mm	2.0 mm
**Control (Blank Formulation)**	3.6 mm	6.6 mm	6.7 mm	7.8 mm	8.6 mm	9.6 mm

### 3.21 Analgesic activity

The results for hot plate method are shown in Table 11. The test formulation exhibited significant effect at 60 min as compared to control. The standard formulation significantly increased the latency period by 12, 15, 17, 18 sec at time 0, 30 min, 60 min and 90 min, respectively, whereas the test formulation significantly increased the licking period (17 sec) at 60 min after that gradually increased (19 s) at 90 min.

**Table 11:** Analgesic activity of blank, standard and test formulation in experimental animals.

**Treatment Groups**	**Licking time (sec) before treatment**				**Licking time (sec) after treatment**			
	**0 min**	**30 min**	**60 min**	**90 min**	**0 min**	**30 min**	**60 min**	**90 min**
**Control (Blank formulation)**	10	11	13	12	11	11	12	12
**Standard Formulation (Voltral® EmulGel 1 %)**	12	13	14	15	12	15	17	18
**Test Formulation (Co-loaded with CAP and TCS)**	11	11	12	14	15	16	17	19

**Notes:** Values are expressed as Mean **±** S.E.M, Analysis was performed with One-Way ANOVA followed by Post Hoc Dunnetts Test.

## 4 Discussion

Cubosomes, a type of vesicular drug delivery system, play a crucial role in delivering drugs precisely to their target sites, ensuring therapeutic effect. These systems incorporate drugs within vesicular structures, enabling targeted and sustained drug delivery. By enhancing the penetration of therapeutic agents across the skin and facilitating their entry into the bloodstream, cubosomes allow efficient delivery of conventional medicines with high molecular weight (Sen et al., 2017). The preparation of nanostructured cubosomal dispersions involved the emulsification of a combination of monoglyceride, surfactant, plus stabilizer in an aqueous medium (Morsi et al., 2014). Our study focused on effectively encapsulating dual analgesic drugs within NCs for synergistic gout therapy. We employed the emulsification method with slight modifications for the formulation. The monoglyceride used in the NC dispersion was glyceryl mono oleate (GMO). GMO is characterized by their biodegradability, non-toxicity, and biocompatibility, owing to their polar lipid composition that closely resembles that of non-ionic surfactants. The hydrocarbon chain, also known as the tail, of oleic acid in GMO has hydrophobic characteristics. On the other hand, the glycerol moiety, or polar head, contains active hydroxyl groups that have the ability to establish hydrogen bonds with the surrounding aqueous medium. As a result, these hydroxyl groups contribute hydrophilic properties to the formulation (Marson et al., 2022). Poloxamers have been widely recognized for their multifunctional properties as solubilizing, wetting, and emulsifying agents (Al-Mahallawi et al., 2021). In this study, poloxamer 407 was utilized as a solubilizer. It is recognized as a self-assembling water-soluble tri-block copolymer. The composition of the material is characterized by the presence of polyethylene oxide (PEO) and polypropylene oxide (PPO) arranged in a conformation known as PEO-PPO-PEO. In this conformation, the hydrophobic qualities are attributed to the PPO portion, while the hydrophilic properties are related to the PEO segment (Dhadwal et al., 2020).

In the current study, zeta potential, particle size, PDI and entrapment efficiency were found to be significantly correlated with the concentrations of GMO, P407 and drugs (both CAP & TCS) in NC formulations. Previous research in the literature has shown that NCs with a GMO: P407 ratio of 9:1 w/w have good physicochemical properties and improve the absorption of poorly absorbed drugs (Jin et al., 2013). Previous findings have shown that varying the GMO concentration between 3.5 % and 5.5 % resulted in the successful production of NC formulations (Khan et al., 2018). Campbell et al demonstrated a study that long chain hydrophilic copolymer significantly led to enhancement in its capability to form smaller particles thus avoiding formation of large aggregates (Campbell et al., 2017). The spreadability of NCs is correlated with a number of variables, such as the retention at the target site, rigidity, length of shear, rate of smearing, and formulation temperature (Alka et al., 2002). The spreadability of all the formulated NC dispersions (F1-F27) was found to be within the range of 2.5 cm to 3.3 cm, which is consistent with the reported data (Chettupalli et al., 2021). Viscosity is important test in determining the drug release from the prepared NC formulation (Archana et al., 2015). The formulated NC formulations (F1-F27) exhibited a pseudo-plastic flow behavior. Under increasing shear stress, the lipid material's (GMO) disordered particles aligned their long axes in the direction of flow. This alignment led to a reduction in the material's internal resistance and, consequently, a decrease in viscosity (El-Enin et al., 2018). The pseudo-plastic flow property ensured the physical stability of the system throughout its shelf-life, facilitated easy application, and improved the retention of the system after application. In our study, we conducted conductivity test which is a crucial method to determine the nature of NC dispersions and identify phase inversion. In our study, the higher conductivity values (ranging from 0.0897 to 0.157 mS/cm) specified that all NC dispersions were o/w type, where water served as the external phase. This higher conductivity is attributed to the larger water phase content, allowing for greater freedom of ion movement (Al-sakini & Maraie, 2019b). The pH values observed in this study were determined to fall within the appropriate range for transdermal formulations. This finding suggests that the risk of skin irritation is minimized and the ability of drugs to pass through the skin is boosted. These results align with earlier research that has demonstrated similar outcomes when ketoconazole is topically applied (Almoshari et al., 2022). In cases where the pH of the prepared formulations exceeded 7, the required pH was achieved using diluted acetic acid (Khan et al., 2022). Over time, slight changes in pH were observed, however the observed variations were taken statistically insignificant (p > 0.05). However, the pH values were observed to decrease from 6.28 to 4.98 after 4 weeks. The drop in pH seen might be attributed to the ionization of the carboxylic groups, which carry a negative charge, present in the free fatty acid components. More precisely, this phenomenon can be related to the ionization of free oleic acid within GMO (Gaballa et al., 2020).

In this study, evaluation of average particle size was performed to ensure that all the particles within the NC dispersion fell within the nanometer size range because it ensures the effective penetration of drugs into the skin layers (Tayel et al., 2016). PDI analysis showed that the NC dispersion with PDI less than 0.45 exhibited good indication of having a broad dispersal of nanoparticles (Tuszynski al., 2021). Similarly the zeta potential imparts indication of good stability. The charge in the nano dispersion was linked to the non-ionic surfactant P407 and the presence of free fatty acid, typically oleic acid, in GMO. These components resulted in a negative charge on the particle surface (Al-sakini et al., 2019a). The relationship between zeta potential and nanoparticle stability was explained using a general guideline. Hoeller et al (2009) stated that zeta potential values within the range of (≤-30 mV) to (≥+30 mV) suggest good stability, whereas values within the range of (≥+60 mV) indicate excellent stability in the formulation. Poloxamer 407, a non-ionic surfactant, has been found to exert its effects through steric stabilization. Zeta potential values equal to or below 20 mV indicate effective stabilization, which can be attributed to the presence of the non-ionic surfactant P407 (Tayel et al., 2016).

In our study, FTIR was also conducted to ensure the compatibility of drugs with the formulation’s excipients. The FTIR profiling of NCs showcases the persistence of all distinctive peaks attributed to CAP, TCS, GMO, and the various constituents within CTNCs. The absence of noticeable structural alterations during the NCs' formulation is evident. Moreover, shifts in peak characteristics, their broadening, or even their disappearance reflect the successful creation of CTNCs, confirming the adept development of NCs co-loaded with CAP & TCS. The preservation of functional group bands within the NCs underscores their consistent nature, with minor shifts, indicating the harmonious compatibility of excipients with the drug entities (Saber et al., 2018). TEM analysis confirmed the physical stability of the nano-cubosomal dispersion which may be attributed to zeta potential on NCs vesicles surfaces leading to repulsion among them (Yosif et al., 2022). We also conducted EE, and capsaicin exhibited higher entrapment efficiency compared to thiocolchicoside. This phenomenon can be attributed to the significant presence of lipid material (GMO) in the formulation. Decreasing the concentration of lipid GMO resulted in a corresponding decrease in capsaicin's entrapment efficiency. The development may be explained by the fact that capsaicin is becoming more soluble in the lipid component of the formulation. Similar results were reported and matched with formulation of nanoparticles loaded with Etoposide from glyceride lipid (Gupta et al., 2012). The higher entrapment efficiency of TCS was due to high hydrophilic nature of thiocolchicoside. Both the GMO/P407 ratio and the TCS content in the formulation have been reported in the literature with improving entrapment efficiency.

We also conducted drug release study and ex vivo permeation analysis because therapeutic efficacy of a drug is proportional to the concentration of drug released from its formulation and permeated through the skin layers. Factors such as lipid GMO, surfactant, co-surfactant, viscosity, and spread ability affect the amount of medication released from a pharmaceutical dosage form (Chaudhary & Sharma, 2021). According to the study conducted by Hosny et al (2022), the GMO composed of lipid material exhibits a significant effect on the retention of drugs inside the layers of the skin, while it also acts as a permeation enhancer. The skin's ability to absorb drugs is affected by various aspects, as demonstrated by an earlier research conducted by Rai et al. (2018). These factors include the basic properties of the drug, the size of the droplets, the zeta potential, and the magnitude of the exposed surface area (Rai et al., 2018). The hot plate model has been employed extensively for the screening of compounds exhibiting analgesia by central mechanism, where elevation in pain threshold of mice towards heat is determined. It is well known fact that, the reaction (paw jumping, licking) by mice to injurious thermal stimuli in hot plate method is supra-spinally mediated response. The analgesic effect exhibited by the test formulation in hot plate test could be due to their interaction with various receptors present in supra-spinal sites. The analgesic effects exhibited that test formulation may be associated with increased licking time and the inhibition of prostaglandins level (Burki et al., 2020).

## 5 Conclusion

In current study, we successfully formulated NCs co-loaded with capsaicin and thiocolchicoside for treatment of inflammation and analgesia usually associated with gout. The most successful NC dispersion attained (F24) exhibited a compact mean particle size, exhibited a negative zeta potential, and maintained a PDI within the optimal range. Additionally, the nearly cubic shape was distinctly revealed through TEM analysis. Both *in vitro* and *in vivo* investigations confirmed the enhanced skin penetration capabilities of NCs co-loaded with capsaicin and thiocolchicoside when applied to rabbit's skin. The NCs exhibited distinct characteristics and a synergistic effect on both anti-inflammatory and analgesic properties. In summary, this study underscores the significance of transdermal co-administered capsaicin and thiocolchicoside using NCs, demonstrating both safety and efficacy in animal models. This approach presents a favorable alternative to oral therapy for individuals with gout and arthritis, and NCs emerge as a promising carrier for enhancing the transdermal delivery of capsaicin and thiocolchicoside, thus offering a synergistic advantage and potentially improving gout management.

## CRediT authorship contribution statement

**Barkat Ali Khan:** Supervision. **Falak Naz:** Conceptualization, Formal analysis, Investigation. **Ali Alqahtani:** Resources, Writing – review & editing. **Muhammad Khalid Khan:** Data curation, Software, Writing – original draft.

## Declaration of competing interest

The authors declare that they have no known competing financial interests or personal relationships that could have appeared to influence the work reported in this paper.
